# Oral Health Care in Europe: Financing, Access and Provision

**DOI:** 10.1093/eurpub/ckac129.372

**Published:** 2022-10-25

**Authors:** J Winkelmann, E van Ginneken, J Gomez Rossi

**Affiliations:** 1 Department of Health Care Management, Berlin University of Technology, Berlin, Germany; 2 European Observatory, Berlin Hub, Berlin, Germany; 3 Oral Health Diagnostics, Charité University, Berlin, Germany

## Abstract

With growing awareness of the large burden of oral diseases and how limited coverage affects both access and affordability, oral health policy has been receiving increased attention in recent years. This culminated in the adoption of the WHO resolution on Oral Health in 2021, which urges Member States to better integrate oral health into their universal health coverage and noncommunicable disease agendas. This study investigates major patterns and developments in oral health status, financing, coverage, access, and service provision of oral health care in 31 European countries. While most countries cover oral health care for vulnerable population groups, the level of statutory coverage varies widely across Europe resulting in different coverage and financing schemes for the adult population. On average, one third of dental care spending is borne by public sources and the remaining part is paid out-of-pocket or by voluntary health insurance. This has important ramifications for financial protection and access to care, leaving many dental problems untreated. Overall, unmet needs for dental care are higher than for other types of care and particularly affect low-income groups. Dental care is undergoing various structural changes. The number of dentists is increasing, and the composition of the health workforce is starting to change in many countries. Dental care is increasingly provided in group practices and by practices that are part of private equity firms. Although there are early signs of a shift towards more prevention of oral diseases, dental care overall remains focused on treatment. A lack of data affects all areas of oral health care and impede to inform policy-making on the underlying causes and the prevalence of oral disease, as well as the effectiveness of community preventive activities and oral health services.

